# Depletion Interactions between Nanoparticles: The Effect of the Polymeric Depletant Stiffness

**DOI:** 10.3390/polym14245398

**Published:** 2022-12-09

**Authors:** Sergei A. Egorov

**Affiliations:** Department of Chemistry, University of Virginia, Charlottesville, VA 22901, USA; sae6z@virginia.edu

**Keywords:** colloids, polymers, semiflexible, phase diagram, density functional theory

## Abstract

A Density Functional Theory is employed to study depletion interactions between nanoparticles mediated by semiflexible polymers. The four key parameters are the chain contour length and the persistence length of the polymeric depletant, its radius of gyration, and the nanoparticle radius. In the Density Functional Theory calculation of the depletion interaction between the nanoparticles mediated by semiflexible polymers, the polymer gyration radius is kept constant by varying the contour length and the persistence length simultaneously. This makes it possible to study the effect of the chain stiffness on the depletion potential of mean force between the nanoparticles for a given depletant size. It is found that the depletion attraction becomes stronger for stiffer polymer chains and larger colloids. The depletion potential of mean force is used as input to compute the phase diagram for an effective one-component colloidal system.

## 1. Introduction

Spherical colloidal particles dispersed in a solution of nonadsorbing polymers play an important role in various systems of biological and technological interest. Accordingly, a detailed understanding of the phase behavior and stability of such dispersions is of primary importance for the development of various industrial applications. As a result, colloidal dispersions in polymer solutions have been actively studied both experimentally [1,2] and theoretically [3,4,5,6]. Despite these extensive studies, several aspects of this problem have remained relatively unexplored. First, most theoretical studies have been limited to fully flexible polymers. At the same time, all realistic polymeric systems involved in numerous practical applications are characterized by some degree of the chain stiffness. Accordingly, the first important research question to be addressed in the present study concerns the effect of the polymer stiffness on the depletion interaction between the colloids. Second, to the best of our knowledge, a direct link between microscopic depletion interaction and phase diagram has not been established yet for semiflexible depletants. Hence, the second research question deals with establishing such a connection by using the depletion interaction calculated from a microscopic model as input for computing a comprehensive colloid–polymer phase diagram. The importance of these two related research questions stems from the fact that a reliable theoretical approach for studying realistic colloid–polymer mixtures and their phase behavior would be of great utility for analyzing the existing experimental data and making suggestions for future experiments. In order to further highlight the novel aspects of the present work and put it in broader context, we next briefly review the existing literature in this field.

From a microscopic perspective, the polymer-induced depletion attraction between two colloidal particles approaching each other in a polymer solution arises from the fact that the chains are expelled from the region between the two colloidal spheres into the bulk due to the loss of configurational entropy by the chains in the region between the spheres. The resulting unbalanced pressure exerted by the polymer chains on the outward surfaces of the two colloids produces an effective depletion attraction between the spheres, which is quantified by the polymer-mediated potential of mean force (PMF) between the two colloids. The knowledge of this PMF makes it possible to treat the colloidal dispersion as an effective one-component system and to construct the corresponding phase diagram describing the stability of the dispersion against flocculation [7,8].

The early pioneering work of Asakura and Oosawa [9,10] developed a theory for the polymer-mediated PMF in colloid–polymer suspensions by treating colloids as hard spheres and polymers as spheres that are fully penetrable to each other but not to the colloids. The resulting PMF produced by the Asakura–Oosawa model is always attractive, with the strength of attraction increasing monotonically with increasing polymer concentration. Following this early work, a large variety of theoretical approaches have been developed to treat colloidal interactions in polymer solutions, including scaling arguments [11], self-consistent field theory [12], and the adsorption method [4] based on the superposition approximation of one-particle depletion layers. In addition, several authors have used the polymer reference interaction site model [13] within the framework of the integral equation theory to compute the PMF in colloid–polymer solutions [14,15,16,17,18,19].

As already mentioned, the vast majority of the aforementioned theoretical studies are limited to fully flexible polymer chains. At the same time, numerous polymeric molecules employed in various practical applications are characterized by a certain degree of stiffness, which is quantified by the chain persistence length [20]. The latter parameter strongly affects the thickness of the polymer depletion layer around a colloid [21], and therefore the polymer-mediated PMF between the colloids [21]. However, both experimental [22,23] and theoretical [16,21,24,25] studies of the chain stiffness effects on the polymer-mediated PMF are still relatively scarce. Among theoretical methods applied to this problem, one can mention integral equation theory [16], self-consistent field theory [21], and density functional theory (DFT) [24,25]. While the former two methods were applied to spherical colloids, the latter approach was limited to the studies of polymer-mediated interactions between flat walls. Accordingly, it would be of interest to apply the DFT formalism to compute the PMF between spherical colloids mediated by semiflexible polymers. The first goal of the present study is to perform such a calculation, with a particular focus on the case when the polymer radius of gyration is comparable to the colloid radius.

As mentioned earlier, the fundamental importance of the polymer-mediated PMF is due to the fact that it allows one to map the colloid–polymer binary mixture onto an effective one-component colloidal system. Once this goal is accomplished, the phase diagram of this one-component system can be computed using standard methods [3]. In the earlier self-consistent field theory study [21] of a binary mixture of spherical colloids and semiflexible polymers, the polymer-induced PMF was employed to compute the second virial coefficient for the colloids, which can be used to characterize the stability of the colloidal dispersion. However, the comprehensive phase diagram of the dispersion has not been obtained directly from he PMF, but rather from the Free Volume Theory [26]. Accordingly, the second goal of the present work is to compute the phase diagram directly from the PMF generated by the DFT approach.

The outline of the remainder of the paper is as follows. In Section 2, we specify our microscopic model; in Section 3, we outline the DFT formalism employed to calculate the polymer-mediated PMF between colloids. In Section 4, we present our approach to obtain the phase diagrams. Section 5 presents our results, and Section 6 concludes the paper.

## 2. Microscopic Model

We consider hard-sphere colloidal particles with radius $R_{c}$ embedded in a solution of semiflexible polymer chains composed of *N* tangent hard sphere beads with diameter σ, i.e., all the bond lengths are fixed at $l_{b}=\sigma$ (σ will be used as the length unit throughout this work). In order to study the effect of chain flexibility on the brush structural properties, we employ a bond-bending potential [27,28]:

(1) $$V_{\mathrm{bend}}\left(\theta_{ijk}\right)=\epsilon_{b}\left[1-\cos\left(\theta_{ijk}\right)\right]=\epsilon_{b}\left[1-\frac{s_{i}\cdot s_{i+1}}{\sigma^{2}}\right],$$

where $\theta_{ijk}$ is the bond angle formed between the two subsequent vectors $s_{i}$ and $s_{i+1}$ along the bonds connecting monomers i,j=i+1 and j,k=i+2, i.e., $s_{i}=r_{i+1}-r_{i}$ and $s_{i+1}=r_{i+2}-r_{i+1}$. The energy parameter $\epsilon_{b}$ then controls the persistence length $l_{p}$, which is defined as [29]

(2) $$l_{p}/l_{b}=-1/\ln\langle \cos\theta_{ijk}\rangle .$$

For semiflexible chains with $\epsilon_{b}\geq 2$, one has the persistence length $l_{p}/l_{b}\approx \beta \epsilon_{b}=\kappa$, where $\beta =1/k_{B}T$ (*T* is the temperature), and κ is the dimensionless stiffness parameter [27,28].

Both colloid–colloid ($v_{cc}\left(r\right)$) and monomer–monomer ($v_{mm}\left(r\right)$) excluded volume interactions are of the hard-sphere type: $v_{cc}\left(r\right)=\infty$ if $r<2R_{c}$ and zero otherwise, and $v_{mm}\left(r\right)=\infty$ if r<σ and zero otherwise. The monomer–colloid interaction is modeled as a sum of hard-sphere repulsion at contact ($v_{mc}\left(r\right)=\infty$ if $r<R_{mc}=0.5\sigma +R_{c}$) and a soft Gaussian repulsion at larger separations: $\beta v_{mc}\left(r\right)=\exp\left[-{\left(r-R_{mc}\right)}^{2}/R_{g}^{2}\right]$, where $R_{g}$ is the polymer chain gyration radius. In order to isolate the effect of the chain stiffness parameter κ on the polymer-induced depletion PMF between the two colloids while keeping the depletant size fixed, we simultaneously vary the chain contour length and its persistence length in such a way that $R_{g}$ remains constant.

## 3. Density Functional Theory

The major goal of the present work is to compute the phase diagram of an effective one-component colloidal system by tracing out the polymeric component. In order to achieve this goal, one needs to compute the polymer-mediated PMF W(R) between the two colloids separated by distance *R*. Combining W(R) with the bare colloid–colloid potential $v_{cc}\left(R\right)$ gives the total interaction $V_{cc}\left(R\right)$ between two colloids:

(3) $$V_{cc}\left(R\right)=v_{cc}\left(R\right)+W\left(R\right).$$

In order to obtain W(R), we define ρ(r,R) as the conditional probability of finding a polymer bead at r given that one colloid is at the origin and the other is located at R (R=|R|). With this definition, the polymer-mediated PMF between the two colloids is given by the following exact relations [14]:

(4) $$W\left(R\right)=\int_{R}^{\infty }F\left(R^{\;\prime }\right)dR^{\;\prime },$$

where the outwards excess mean force, F(R), is given by:

(5) $$F\left(R\right)=-\int dr(\nabla v_{mc}\left(r\right)\cdot \hat{R})\rho \left(r,R\right),$$

where $\hat{R}$ is the unit vector along the line connecting the two colloids, and ρ(r,R) is the anisotropic monomer density profile induced by the two colloids. In the present study, we construct the latter density profile on the basis of the Kirkwood superposition approximation (KSA) [8], whereby ρ(r,R) is approximated by the product of spherically symmetric density profiles ρ(r) around individual colloids: $\rho_{KSA}\left(r,R\right)\approx \rho \left(r\right)\rho (|r-R|)/\rho$, with ρ being the bulk monomer density.

The isotropic monomer density profile ρ(r) around a single colloid is obtained from the DFT formalism [30,31,32]. As a starting point of the DFT-based approach, one writes an expression of the grand free energy, Ω, as a functional of the polymer density profile $\rho_{p}\left(R_{p}\right)$, where $R_{p}=\left(r_{1},r_{2},\cdots ,r_{N}\right)$ is a collective variable with the individual monomer coordinates $r_{i}$. The average monomer density ρ(r) is related to the molecular density profile, $\rho_{p}\left(R_{p}\right)$, as follows:

(6) $$\rho \left(r\right)=\int dR_{p}\sum_{i=1}^{N}\delta \left(r-r_{i}\right)\rho_{p}\left(R_{p}\right)$$

The minimization of Ω with respect to $\rho_{p}\left(R_{p}\right)$ yields the equilibrium polymer density distribution. The functional Ω is related to the Helmholtz free energy functional, *F*, via a Legendre transform:

(7) $$\Omega \left[\rho_{p}\left(R_{p}\right)\right]=F\left[\rho_{p}\left(R_{p}\right)\right]+\int dR_{p}\rho_{p}\left(R_{p}\right)\left[V_{ext}\left(R_{p}\right)-\mu \right],$$

where μ is the polymer chemical potential, and $V_{ext}\left(R_{p}\right)$ is the external field, which in the present case is due to the interaction of the polymer beads with the colloid:

(8) $$V_{ext}\left(R_{p}\right)=\sum_{i=1}^{N}v_{mc}\left(r_{i}\right).$$

We employ the following approximation for the Helmholtz free energy functional, which separates it into ideal and excess parts according to [33]:

(9) $$F\left[\rho_{p}\left(R_{p}\right)\right]=F_{\mathrm{id}}\left[\rho_{p}\left(R_{p}\right)\right]+F_{\mathrm{ex}}\left[\rho \left(r\right)\right],$$

with the ideal functional given by [34,35]:

(10) $$\beta F_{\mathrm{id}}\left[\rho_{p}\left(R_{p}\right)\right]=\int dR_{p}\rho_{p}\left(R_{p}\right)\left[\ln\rho_{p}\left(R_{p}\right)-1\right]+\beta \int dR_{p}\rho_{p}\left(R_{p}\right)V_{b}\left(R_{p}\right)+\beta \sum_{i=1}^{N-2}\int dR_{p}\rho_{p}\left(R_{p}\right)V_{\mathrm{bend}}\left(s_{i},s_{i+1}\right),$$

where $V_{\mathrm{bend}}$ is given by Equation (1), and $V_{b}\left(R_{p}\right)$ is the binding energy given by [36]:

(11) $$\exp\left[-\beta V_{b}\left(R_{p}\right)\right]=\prod_{i=1}^{N-1}\frac{\delta (|r_{i}-r_{i+1}|-\sigma )}{4\pi \sigma^{2}}=\prod_{i=1}^{N-1}g_{b}\left(|r_{i}-r_{i+1}\right).$$

For the excess free energy functional, we adopt the weighted density approximation [37]:

(12) $$\beta F_{\mathrm{ex}}\left[\rho \left(r\right)\right]=\int dr\rho \left(r\right)f_{\mathrm{ex}}\left(\bar{\rho }\left(r\right)\right),$$

with the weighted density given by:

(13) $$\bar{\rho }\left(r\right)=\int dr^{\prime }\rho \left(r^{\prime }\right)w(|r-r^{\prime }\left|\right).$$

In the above, the monomer density ρ(r) is given by Equation (6), and $f_{\mathrm{ex}}\left(\rho \right)$ is the excess free energy density per site of the polymer solution with site density ρ arising from the short-ranged hard-core repulsive interactions. We compute it from the Wertheim’s expression which was obtained on the basis of the first-order thermodynamic perturbation theory [38]:

(14) $$f_{\mathrm{ex}}\left(\rho \right)=\frac{4\eta -3\eta^{2}}{{\left(1-\eta \right)}^{2}}-\left(1-\frac{1}{N}\right)\ln\frac{1-\eta /2}{{\left(1-\eta \right)}^{3}}$$

where $\eta =\pi \sigma^{3}\rho /6$ is the monomer packing fraction.

In the present work, we employ the simple square-well form for the weighting function w(r), whose range is given by the diameter σ of the polymer segment [39]:

(15) $$w\left(r\right)=\frac{3}{4\pi \sigma^{3}}\Theta \left(\sigma -r\right),$$

where Θ(r) is the Heaviside step function. While more sophisticated forms of the weighting function are available in the literature (e.g., those used in the Fundamental Measure Theory version of DFT [40]), earlier studies [41] have shown relative insensitivity of DFT results for polymeric systems to the specific choice of the weight function.

The minimization of the grand free energy functional Ω yields the following result for the equilibrium polymer density profile [32]:

(16) $$\rho_{p}\left(R_{p}\right)=\prod_{i=1}^{N-1}g_{b}\left(|r_{i}-r_{i+1}\right)\prod_{i=1}^{N-2}\exp\left[-\beta V_{\mathrm{bend}}\left(s_{i},s_{i+1}\right)\right]\prod_{i=1}^{N}\exp\left[-\lambda \left(r_{i}\right)\right],$$

where

(17) $$\lambda \left(r\right)=\beta \frac{\delta F_{ex}}{\delta \rho (r)}+\beta v_{mc}\left(r\right).$$

Substitution of $\rho_{p}\left(R_{p}\right)$ into Equation (6) then yields an integral equation for the monomer density distribution ρ(r) which needs to be solved numerically [32].

Regarding the numerical implementation of the DFT procedure, given that the monomer density distribution around a single colloid is spherically symmetric, the corresponding integral equation for ρ(r) is solved numerically on an equidistant grid along the radial coordinate *r* with the grid spacing Δr=0.02. A simple Picard iteration procedure was employed [42], and tolerance criterion for terminating the iterative procedure was set to $10^{-6}$.

## 4. Phase Diagrams

In order to calculate the phase diagram, we follow the standard approach [3,8] and map the two-component colloid–polymer mixture onto an effective one-component colloidal system by tracing out the polymeric component. This goal is accomplished via Equation (3), whereby the bare hard-sphere colloid–colloid potential $v_{cc}\left(r\right)$ is augmented by the polymer-mediated depletion PMF W(R) obtained from Equation (4) using DFT formalism outlined in Section 3. In calculating the phase diagram, we consider both fluid (vapor and liquid) and solid phases. The phase boundaries are obtained by equating the pressure and the colloid chemical potential in the two coexisting phases [3,8].

The dimensionless pressure of a fluid phase is given by [3]:

(18) $$\frac{PV_{c}}{k_{B}T}=\frac{\eta_{c}+\eta_{c}^{2}+\eta_{c}^{3}-\eta_{c}^{4}}{\left(1-\eta_{c}^{3}\right)}+\frac{\rho_{c}\eta_{c}}{2}\int dr\beta W\left(r\right),$$

where $V_{c}=4\pi R_{c}^{3}/3$ is the volume of the colloidal sphere, $\rho_{c}$ is the colloid number density, and $\eta_{c}=\rho_{c}V_{c}$ is the colloid packing fraction. The first term on the right-hand side of Equation (18) originates from the Carnahan–Starling equation of state [43], while the second term comes from the mean-field treatment of the effective colloid–colloid attraction due to the polymer-mediated depletion interaction.

The dimensionless chemical potential of a fluid phase is given by [3]:

(19) $$\frac{\mu }{k_{B}T}=\ln\frac{\Lambda_{c}^{3}}{V_{c}}+\ln\eta_{c}+\frac{3-\eta_{c}}{\left(1-\eta_{c}^{3}\right)}-3+\rho_{c}\int dr\beta W\left(r\right),$$

where $\Lambda_{c}$ is the de Broglie wavelength of the colloidal particle.

The dimensionless pressure of a solid phase is given by [3]:

(20) $$\frac{PV_{c}}{k_{B}T}=\frac{3\eta_{c}}{1-\eta_{c}/\eta_{cp}}+\frac{\rho_{c}\eta_{c}}{2}\int dr\beta W\left(r\right),$$

where $\eta_{cp}=\pi /\left(3\sqrt{2}\right)$ is the value of $\eta_{c}$ at close packing.

Finally, the dimensionless chemical potential of a solid phase is given by [3]:

(21) $$\frac{\mu }{k_{B}T}=\ln\frac{\Lambda_{c}^{3}}{V_{c}}+\frac{27}{8\eta_{cp}^{3}}+3\ln\left[\frac{\eta_{c}}{1-\eta_{c}/\eta_{cp}}\right]+\frac{3}{1-\eta_{c}/\eta_{cp}}+\rho_{c}\int dr\beta W\left(r\right),$$

## 5. Results

In order to focus on the effect of the polymer stiffness parameter κ on the colloid–colloid polymer-induced depletion interaction and the corresponding phase behavior of the effective one-component colloidal system, we start by computing the polymer chain radius of gyration $R_{g}$ for a range of values of the chain contour length *N* and stiffness parameter κ. The radius of gyration for our microscopic model of the semiflexible chain specified in Section 2 is calculated using the DFT methodology described in detail in Ref. [44] (with the Helmholtz free energy functional and the weighted density defined in Section 3). All the results reported below were obtained for the value $R_{g}=10$ and the following five pairs of values of *N* and κ: (N=40, κ = 25.1), (N=48, κ = 11.5), (N=64, κ = 6.2), (N=80, κ = 4.4), and (N=96, κ = 3.5). While in the first pair the contour and persistence lengths are comparable, in the last pair $l_{p}$ is nearly two orders of magnitude smaller than contour length. Thus, these selected values span the range from semiflexible to nearly fully flexible polymeric depletants (at the fixed depletant size).

**Figure 1:**
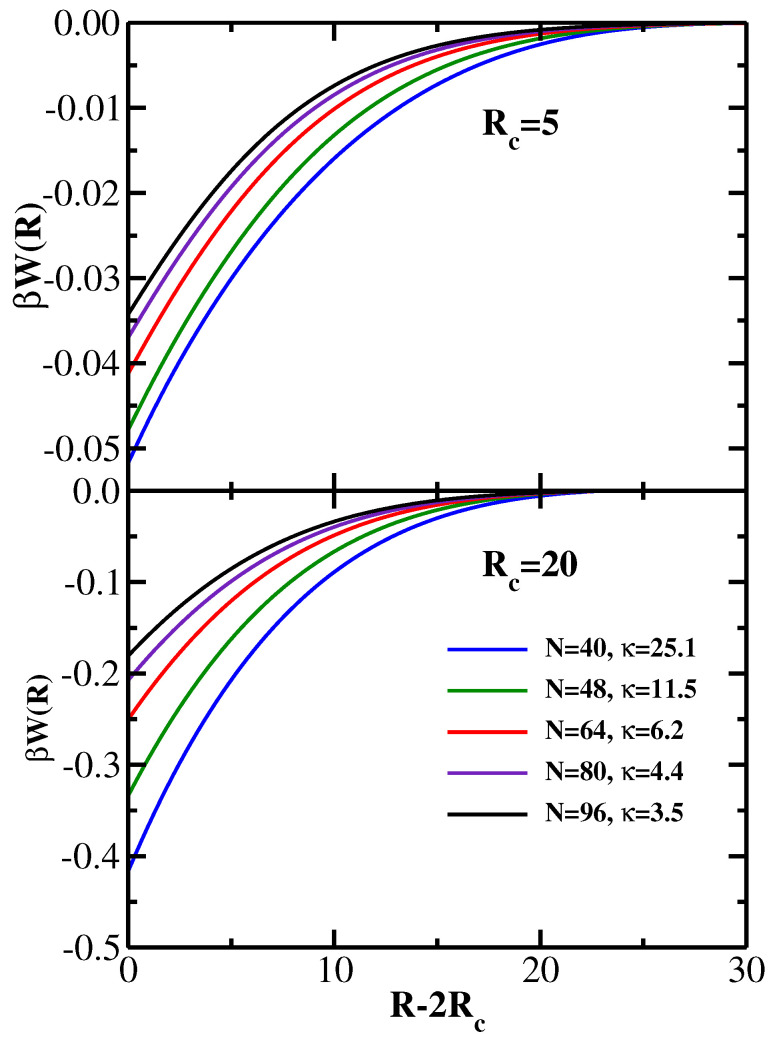
Dimensionless colloid−colloid βW(R) as a function of colloid separation $R-2R_{c}$ for five values of the stiffness parameter κ as indicated in the legend. The monomer bulk density is fixed at ρ=0.001. Upper panel: colloid radius $R_{c}=5$; Lower panel: colloid radius $R_{c}=20$.

Using the DFT approach, we compute the polymer-induced PMF for five pairs of values of *N* and κ listed above; the monomer bulk density is fixed at ρ=0.001. The DFT results for the dimensionless PMF βW(R) are presented in Figure 1 for two values of the colloid radius: $R_{c}=5$ in the upper panel, and $R_{c}=20$ in the lower panel. Thus, the former case corresponds to the situation $R_{c}<R_{g}$, while in the latter case has $R_{c}>R_{g}$. One sees that for both values of the colloid radius the strength of the depletion attraction and its range increase with increasing chain stiffness, in agreement with earlier SCF results [21]. The depletion attraction also becomes stronger with increasing colloid radius, as one would expect [21].

**Figure 2:**
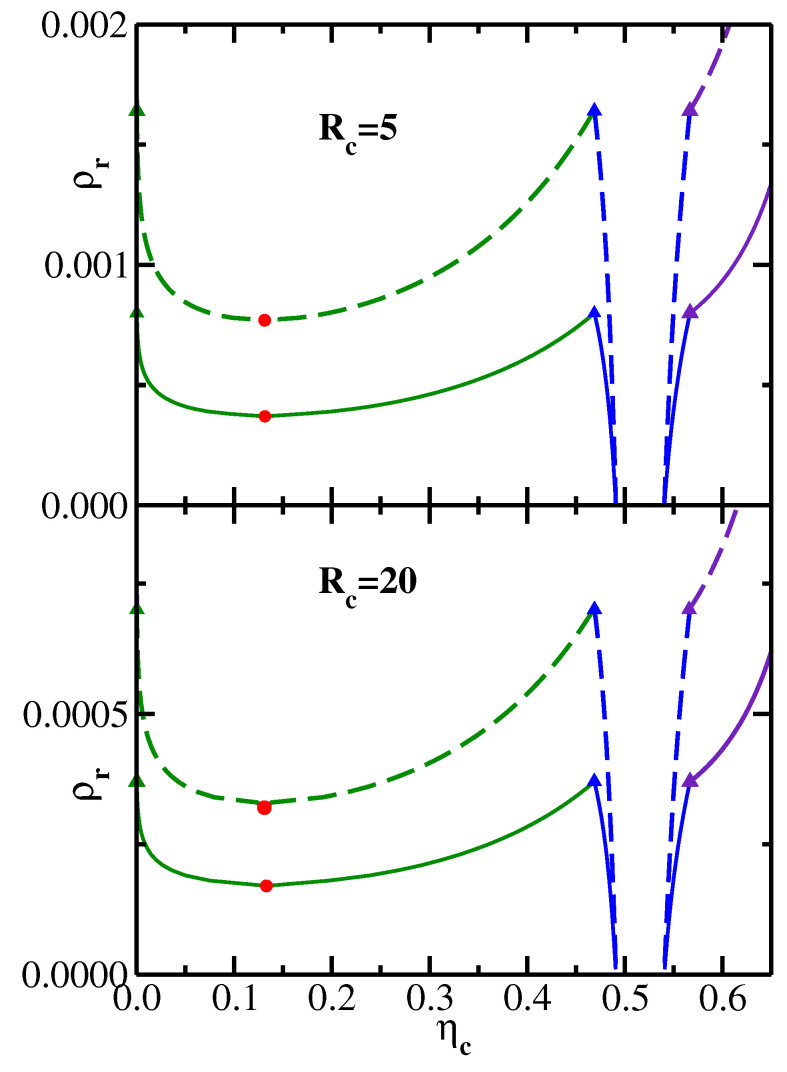
Phase boundaries for an effective one-component colloidal system in the reservoir representation in the variables $\eta_{c}$–$\rho_{r}$. Green lines denote the liquid–vapor phase boundary, blue lines mark the liquid–solid phase boundary, and purple lines correspond to the vapor–solid phase boundary. Red circles mark the location of the liquid–vapor critical points, and the triangles denote the vapor–liquid–solid triple point coexistence. Upper panel: solid lines correspond to the polymeric chains with (N=40, κ = 25.1); dashed lines correspond to the polymeric chains with (N=96, κ = 3.5); the colloid radius is $R_{c}=5$. Lower panel: solid lines correspond to the polymeric chains with (N=48, κ = 11.5); dashed lines correspond to the polymeric chains with (N=96, κ = 3.5); the colloid radius is $R_{c}=20$.

In order to compute the phase diagrams of the effective one-component colloidal systems, we obtain the depletion PMFs for a range of values of the reservoir monomer density $\rho_{r}$ [26] and then use these results to calculate the phase boundaries as described in Section 4. Some representative results in the variables $\eta_{c}$–$\rho_{r}$ (reservoir representation [26]) are shown in Figure 2, where the upper panel corresponds to the colloid radius $R_{c}=5$, and the lower panel corresponds to the colloid radius $R_{c}=20$. In the upper panel, the solid lines correspond to the semiflexible polymeric depletant with N=40 and κ = 25.1, while the dashed lines correspond to more flexible chains with N=96 and κ = 3.5. In the lower panel, the solid phase boundary lines correspond to the system (N=48, κ = 11.5) and the dashed lines to the system (N=96, κ = 3.5). In both panels, the circles mark the location of the liquid–vapor critical points, while the triangles denote the vapor–liquid–solid triple point coexistence. In the reservoir representation, the triangles marking these three coexisting phases all correspond to the same value of the monomer reservoir density $\rho_{r}$ and therefore lie on a horizontal line (all tie-lines connecting coexisting phases are horizontal in the reservoir representation). As one would expect from the PMF results presented in Figure 1, the phase boundaries move to lower values of $\rho_{r}$ with increasing chain stiffness (solid lines lie below dashed lines in both panels of Figure 2). Likewise, for a given polymeric depletant (N=96, κ = 3.5), the phase boundaries move to lower values of $\rho_{r}$ with increasing colloid radius, as can be seen by comparing the dashed lines in the upper and lower panels of Figure 2.

**Figure 3:**
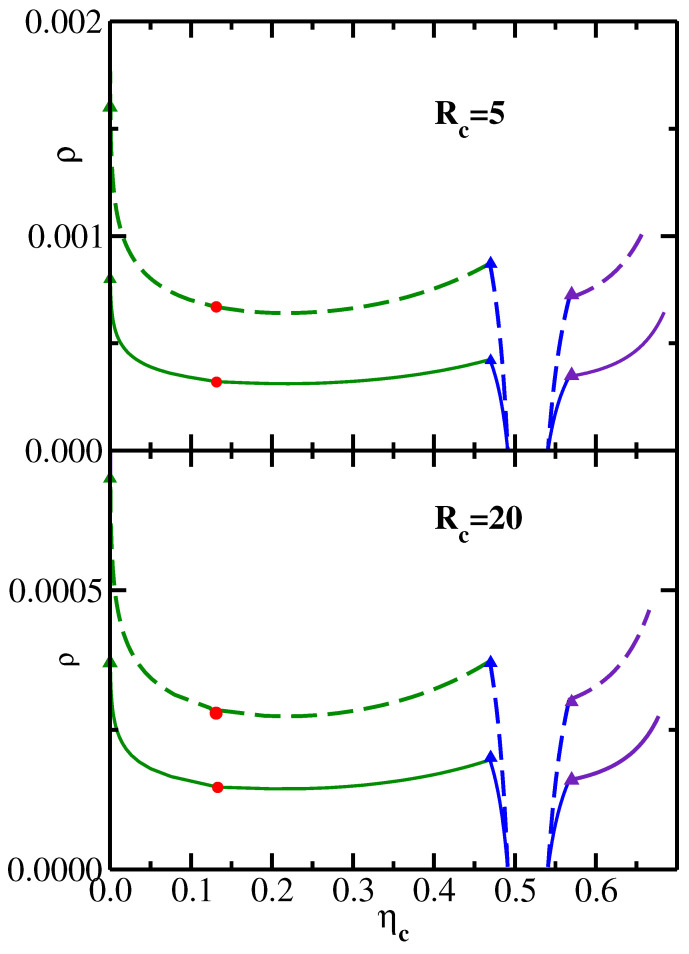
Same as Figure 2 but in the system representation in the variables $\eta_{c}$–ρ.

Figure 3 replots the data shown in Figure 2 in a system representation [26] in the variables $\eta_{c}$–ρ. The transformation from the reservoir to the system representation is achieved [3] via the equation $\rho =\rho_{r}\left(1-\eta_{c}\right)$. It is important to note that the range of ρ values in Figure 3, as well as the range of $\rho_{r}$ values in Figure 2, are both sufficiently low, so that no isotropic–nematic transition of semiflexible polymers needs to be taken into account [27,28].

**Figure 4:**
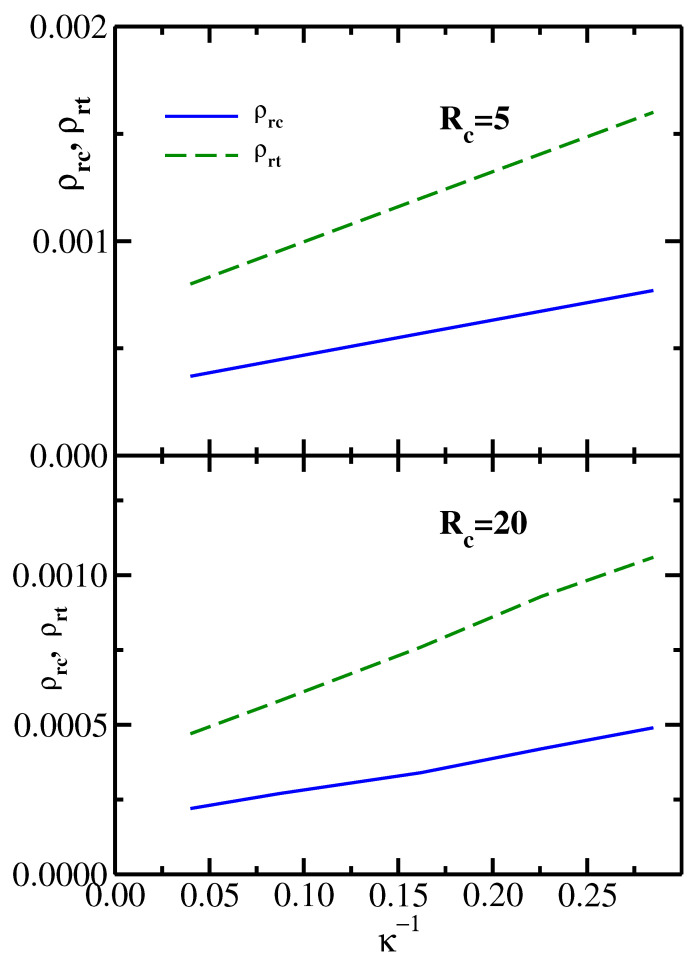
The monomer reservoir density corresponding to the liquid–vapor critical point $\rho_{rc}$ and to the vapor–liquid–solid triple point $\rho_{rt}$ as functions of the inverse stiffness parameter $\kappa^{-1}$. Upper panel: colloid radius $R_{c}=5$; Lower panel: colloid radius $R_{c}=20$.

It follows from the results shown in Figure 2 that the monomer reservoir density $\rho_{rc}$ (which corresponds to the liquid–vapor critical point), as well as $\rho_{rt}$ (the monomer reservoir density corresponding to the vapor–liquid–solid triple point), both decrease with increasing chain stiffness κ. This behavior is further illustrated in Figure 4, which plots both $\rho_{rc}$ and $\rho_{rt}$ as functions of the inverse stiffness parameter $\kappa^{-1}$. The upper panel presents the results for the colloid radius $R_{c}=5$, while the lower panel shows the results for the colloid radius $R_{c}=20$. For both values of the colloid size, $\rho_{rc}$ and $\rho_{rt}$ increase nearly linearly with $\kappa^{-1}$. Furthermore, the slope of the triple point line $\rho_{rt}$ is significantly higher than the slope of the critical point line $\rho_{rc}$. This behavior is indeed consistent with the phase diagrams shown in Figure 2, where one sees that an increase in chain stiffness (going from dashed to solid phase boundaries) leads to a substantially larger drop in $\rho_{rt}$ (marked by triangles) compared to the drop in $\rho_{rc}$ (marked by circles).

**Figure 5:**
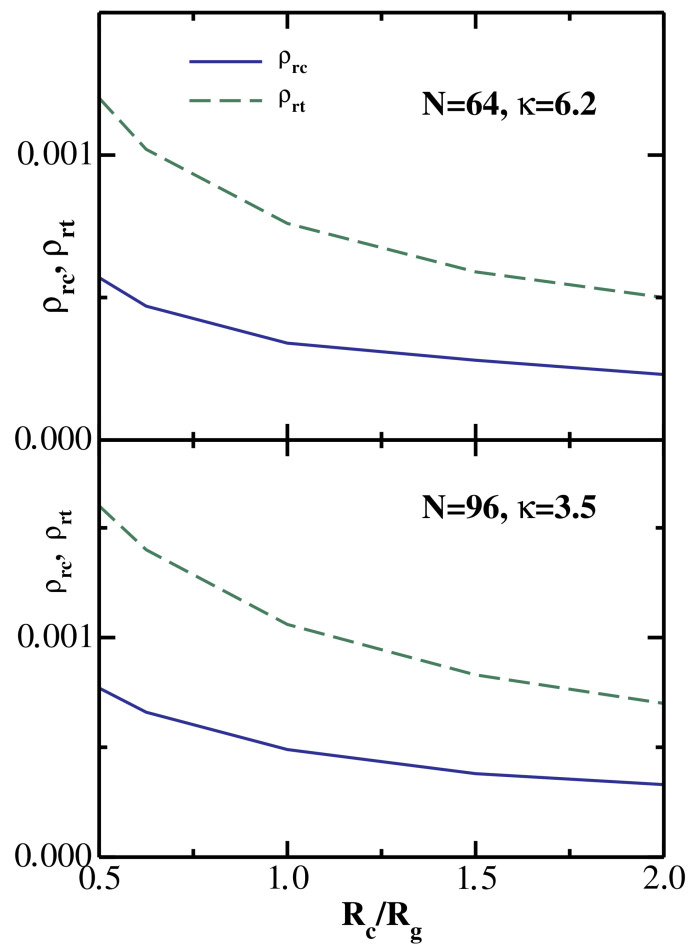
The monomer reservoir density corresponding to the liquid–vapor critical point $\rho_{rc}$ and to the vapor–liquid–solid triple point $\rho_{rt}$ as functions of the ratio $R_{c}/R_{g}$. Upper panel: semiflexible chain with (N=64, κ=6.2); Lower panel: semiflexible chain with (N=96, κ=3.5).

By comparing the upper and lower panels of Figure 2, one sees that both $\rho_{rc}$ and $\rho_{rt}$ decrease with increasing colloid radius, which is consistent with W(R) becoming more attractive for larger colloids, as shown in Figure 1. The dependence of the critical and triple reservoir monomer densities on the dimensionless ratio $R_{c}/R_{g}$ is illustrated in Figure 5 for two particular semiflexible chains: (N=64, κ = 6.2) in the upper panel and (N=96, κ = 3.5) in the lower panel. One sees that for both depletants $\rho_{rc}$ and $\rho_{rt}$ decrease monotonically with increasing $R_{c}/R_{g}$, as one would expect based on the results shown in Figure 1 and Figure 2.

## 6. Conclusions

In this work, we have developed a Density Functional Theory for the depletion potential of mean force between spherical colloids induced by semiflexible polymer chains. The theory was used to study the effects of the colloid radius (relative to the polymer radius of gyration) and the chain stiffness (for a given value of $R_{g}$) on the strength of the depletion attraction. In agreement with earlier self-consistent field theory calculations [21], it was found that depletion attraction becomes stronger for larger colloids and stiffer chains.

The colloid–colloid potential of mean force was calculated for a range of monomer densities, and the results were subsequently used to construct phase diagrams for an effective one-component colloidal system, both in the reservoir and in the system representations of the monomer density. The phase boundaries for vapor–liquid, vapor–solid, and liquid–solid phase coexistence were obtained, as well as vapor–liquid critical points and vapor–liquid–solid triple points. The reservoir monomer densities corresponding to critical and triple point were found to increase nearly linearly with inverse chain stiffness, with the slope of the former being substantially smaller than the latter.

This work can be extended in several important directions. First, it would be of interest to study the morphology of the crystalline phase. To achieve this, the microscopic model must be made more chemically specific in order to enable comparison with existing experimental data obtained by tunneling electron microscopy. Second, given the importance of photocatalytic activity for practical applications of these systems, one could combine the present results with quantum DFT calculations with a goal of estimating the photocatalytic activity and comparing it with previous studies. These directions will be the subjects of future research.

## Data Availability

The data that support the findings of this study are available from the corresponding author upon reasonable request.
